# Leadership and *Karma*: doing good or doing well?

**DOI:** 10.1192/bjb.2024.107

**Published:** 2025-12

**Authors:** Swaran P. Singh

**Affiliations:** University of Warwick, Coventry, UK.

**Keywords:** History of psychiatry, mental health services, philosophy, transcultural psychiatry, clinical governance

## Abstract

Modern management has much to learn from ancient wisdoms. Management structures based on corporate trends were transferred from business to services such as healthcare to promote cost-efficiency and productivity. In this article, I argue that the short-term approach of corporate leaders being brought into healthcare for ‘transformation’ has led to a trail of service dismemberment with no discernible clinical gain for those we seek to serve. *Bhagwad Gita*, the ancient Hindu scripture on right conduct, is an exemplar of how the primary aim of leaders should be to provide better service rather than serve personal interests or those of the ‘business’ of healthcare.

## The past is another country

Thomas Adeoye Lambo (1923–2004) was the first Western-trained psychiatrist in Nigeria and possibly Africa.^1^ He transformed the Aro Neuropsychiatric Hospital in Abeokuta from an asylum to a thriving community enterprise where patients, while being treated, were integrated into the local community. Professor Lambo is buried in the grounds of the Aro hospital. It is difficult to imagine the chief executive of a contemporary psychiatric service given such honour or evoking similar love, pride and respect.

Medical superintendents of long-stay hospitals devoted long periods of their lives to the institutions they served. In the UK, Thomas Percy Rees (1899–1963)^2^ became Medical Superintendent of Warlingham Park Hospital in 1935, having served as Deputy Medical Superintendent from 1927. Other examples of individuals with long tenures are Duncan Mapperley, Medical Superintendent in Nottingham for 27 years, David Clarke at Fulbourn Hospital for 30 years, Angus Mackay at Argyll and Bute Hospital for 24 years, and Alexander Walk at Cane Hill for 23 years (A. MacKay, personal communication, 2024). These leaders made key decisions and stayed in their roles long enough to see the impact of those decisions. They had, to use a popular phrase, ‘skin in the game’. The role of a single medical leader ended with the Nodder report (1980), which was commissioned because of multiple failures and scandals in large asylums.

In this article, I argue that the current management structure, with chief executive officers (CEOs) appointed on short-term tenures and judged on cost-related ‘efficiencies’, is inimical to both patient and National Health Service (NHS) interest. Using the example of a moral dilemma from the Indian scripture *Bhagwad Gita*, I suggest that the personal interests of NHS leaders and managers (‘doing well’) should be subservient to what is best for patients and the NHS (‘doing good’). The aim is not to proselytise, glorify one culture at the expense of another, or nostalgically hanker for the past when doctors were all-powerful. This is a humble reflection on what can still be learned from ancient wisdom.

## Current NHS management

Beginning with the Griffiths Report (1983),^3^ NHS management has undergone a series of changes, with debates on whether NHS is overmanaged, undermanaged or inappropriately managed.^4^ This paper is not a review of changes in NHS management since its creation or of the underlying political drivers. I believe that the zero-sum nature of contemporary political debate – where for one side to be right, the other must necessarily be wrong – has driven successive governments to impose ideological changes for short-term political expediency. The NHS, like education, is a victim of short-termism,^5^ which itself is a cause of long-term decline. Health and education should be too important to be political; radically changing these every few years on ideological grounds damages their fabric and ethos. A mature political system should recognise that complex problems are not binary. Improvements in large complex systems are most likely to succeed when they are incremental, allowed time to bed in and carefully evaluated. This paper simply contrasts modern managers with earlier medical superintendents and suggests that today's rapid turnover of senior leaders creates perverse incentives.

Although contemporary NHS CEOs are appointed on 5-year contracts, their median tenure is 3 years (with a mean of 4 years).^6^ Any new CEO will need a minimum of 18 months to get to grips with the role and understand the intricacies of the organisation, its functioning, and its financial health and outlook. As the clock ticks on their tenure, the CEO begins searching for a new job at the start of their fourth year. This gives a CEO 18–24 months for any demonstrable achievement, which is necessary to secure the next role.

What can any CEO achieve in under 2 years? They can reorganise the structure and create a series of new teams and managers to deliver a strategic vision with mission statements, business plans, communication and media strategies, integration, technology and IT changes, audits, risk, learning culture, equality and diversity, and other reshuffling that may or may not be relevant to patient outcomes. Service transformations become bi- or triennial rituals that demand alterations in care provision, without identifying the need for change. Platitudes and cliches – learning lessons, equity, inclusion, level playing-field, at the front line, being in the driving seat, reinventing the wheel, one size fits all, etc. – are thrown about, taskforces are created, stakeholder meetings arranged, frameworks designed and services transformed. The impact, positive or deleterious, is never measured, as the changes last only as long as the next CEO appointment.

Having reorganised structures with no measurable improvement, the CEO has limited options. Canny CEOs will manage to find friendless services, such as day hospitals or respite provisions, that can be abolished with promises of enhanced community care. The CEO thereby achieves cost reduction. Demolition is instantaneous; with a single stroke of a pen, thousands of pounds can be ‘saved’. Worse still is dismembering an existing excellent service and redistributing its resources to poorly performing ones, with the stated aim of equity. The CEO can now claim transformative leadership and move to another organisation.

Starting a new service or genuinely improving an existing one is a long-term endeavour; going from the inception of an idea to achieving demonstrable improvements is like building a garden on parched land. It requires years of hard work, dedication, commitment and resources. Few CEOs have the continuity of tenure to see genuine improvements through to maturity. Doing good for patients becomes secondary to doing well for oneself as CEOs become adept at showing cost-efficiency.

## Doing good or doing well?

Doing good and doing well need not be in conflict, provided that ‘doing’ is not driven purely by personal interests. As an illustration, I use advice from Hindu scripture *Bhagwad Gita* which deals extensively with conflict between self-interest and altruism.

*Bhagwad Gita* (literally divine song) is a dialogue between the warrior Arjuna and his charioteer Lord Krishna. It is part of one of the longest and oldest religious epics in the world, *Mahabharata*, which describes a war between two clans of cousins, the *Pandavas* and *Kauravas*. At the beginning of the war, as the two armies face each other on the battlefield, Arjuna, one of the *Pandavas*, describes his anguish at having to kill people who have been his friends, teachers and elders. Krishna tells Arjuna that the duty of a warrior is to fight injustice; hence, war is righteous action. Krishna argues that when deciding to act, one should be driven not by self-interest but by concern about the well-being of others. In this war, Arjuna's duty to fight injustice should override his personal anguish.

Such righteous conduct – *Nishkam Karma* (literally desireless or selfless action; altruism is the closest English term) *–* is a salient feature of Indian religions. It is not a purposeless action without a thought or motive. Instead, it is a requirement to act righteously in accordance with ethical principles. *Karma* is not, as commonly assumed, a signifier of passive acceptance of fate or destiny but an imperative that a being with agency should act in accordance with *Dharma* (virtuous conduct) and with a dispassionate attitude towards personal consequences.^7^

This is not a cynical view of managers, nor is it an ungracious attack on hard-working colleagues who lead large and complex organisations facing multiple challenges, including dwindling resources. However, over 30 years in the NHS, I have personally witnessed – as a clinician and a carer – trails of service dismemberment by management chasing short-term ‘gains’.

It is easy to glorify the past. Past medical superintendents were very likely to have been driven by factors other than altruism. However, these superintendents stayed in leadership positions long enough to see the consequences of their actions and the impact on patient care. The contemporary CEO is not a bad person or necessarily a poor leader; the demands of the system, especially around costs and efficiencies, incentivise their actions differently, and the short tenure means they never stay long enough to be accountable.

If all management decisions were to be based on a single premise – is this the right action for patient welfare? – the NHS might be in a better state. Leadership that is based on servitude rather than control, on self-reflection rather than reflex expediency, on ‘rightness’ of the action rather than cost implications, may have created a very different NHS from the one we currently have. Good leadership is not about the profession of the leader as much as their intrinsic motivation, their commitment to patient care and their long-term vision. The current system, with its top-heavy complex hierarchies, staff churn, constant service change and focus on short-term gains, means that accountability falls only to clinicians, who are often least involved in these transformations. A good place to improve would be to ensure that those leading transformations are held accountable for their impacts, even if individuals have moved to another organisation.

## Focus on right action

I have argued that ancient wisdom such as *Nishkam Karma* has much to teach contemporary managers. I do not intend to moralise or indulge in pious posturing. I am aware of the resource pressures on the NHS. However, I have witnessed repeated ‘reform’ of the NHS based on political and economic imperatives rather than a focus on right action. The solution to misalignment of incentives with performance might be creating incentives that better correspond to demonstrable service improvement rather than simply service change; and CEO performance should be evaluated against not just short-term activity but longer-term improvement in patient care and outcomes. In this cynical age, appealing to virtue may not convince either service providers or senior leaders. I am arguing, however, that virtuous conduct still has a role in public service. Within the NHS, a leader's concern should be about patient care rather than career advancement, and to focus on the right action rather than a personally desirable outcome. I have selected a message from *Bhagwad Gita*: one is likely to find the same theme repeated in many other theological and philosophical traditions.

## About the author

**Swaran P. Singh** is at Warwick Medical School, University of Warwick, Coventry, UK.
